# Supplementary material for: Frizzled2 receives WntA signaling during butterfly wing pattern formation
Source: Development. 2023 Sep 28;150(18):dev201868. doi: 10.1242/dev.201868 (PMC10560568; doi:10.1242/dev.201868)
Supplement: Supplementary information [file develop-150-201868-s1.pdf]

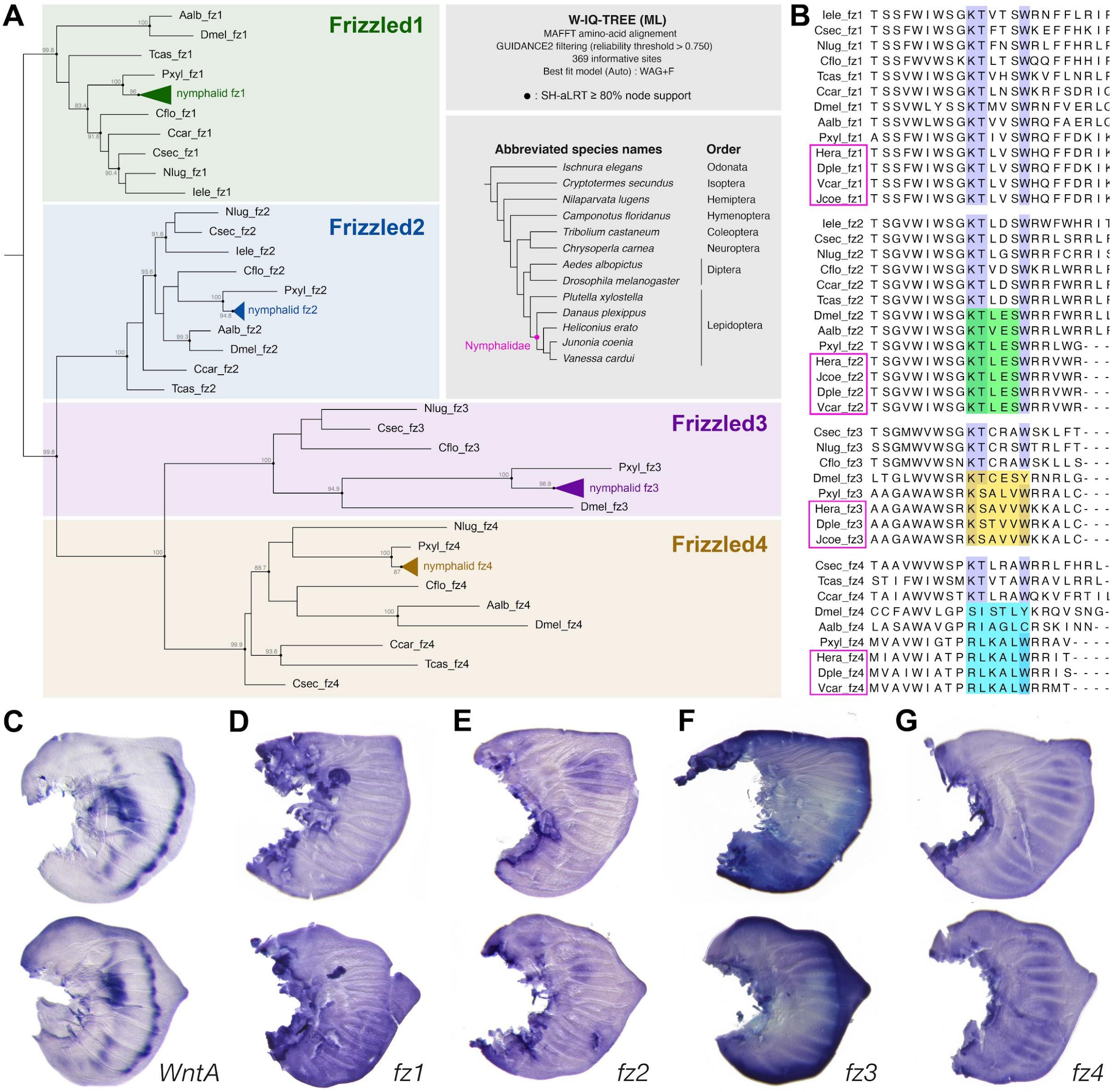

Fig. S1. Four Frizzled-family receptors in Nymphalidae. (A) Phylogenetic reconstruction of insect Frizzled family receptors (source sequences and accession numbers in Supplementary File 1). Nymphalid butterflies encode 4 *frizzled* genes belonging to pre-Bilaterian orthology groups (Schenkelaars *et al.* 2015). **(B)** Alignment of insect Frizzled genes spanning the end of the TM7 domain (positions 1-7) and the beginning of the C-terminal cytoplasmic region (positions 8-23). Magenta : nymphalid butterfly sequences. Blue : conserved KTxxxW motif necessary for interaction with Dsh. Green : both lepidopteran and dipteran genomes show a conserved KTLES motif, needed for the Dsh-independent FNI transduction of Fz2 in *Drosophila* (Matthew *et al.* 2005). Yellow : the Fz3 KTxxxW motif is degenerated inDiptera, while lepidopteran orthologues evolved a Thr>Ser substitution. Cyan : the KTxxxW motif is absent from lepidopteran and dipteran copies of Fz4. **(C-G)** ISH of mRNA probes in *V. cardui* 5th instar larval imaginal disks for forewings (top) and hindwings (bottom). **(C)** *WntA* shows strong expression in the presumptive CSS and MBS of larval wing discs. **(D)** *fz1* shows low ubiquitous expression throughout the imaginal disk. **(E)** *fz2* shows low overall expression with increased expression anterior of the M_3 vein. **(F)** *fz3* shows intense staining in the *wg-Wnt6-Wnt10* expressing peripheral tissue, with weaker expression visible in the wing epithelium. **(G)** *fz4* shows interveinous expression.

Development • Supplementary information

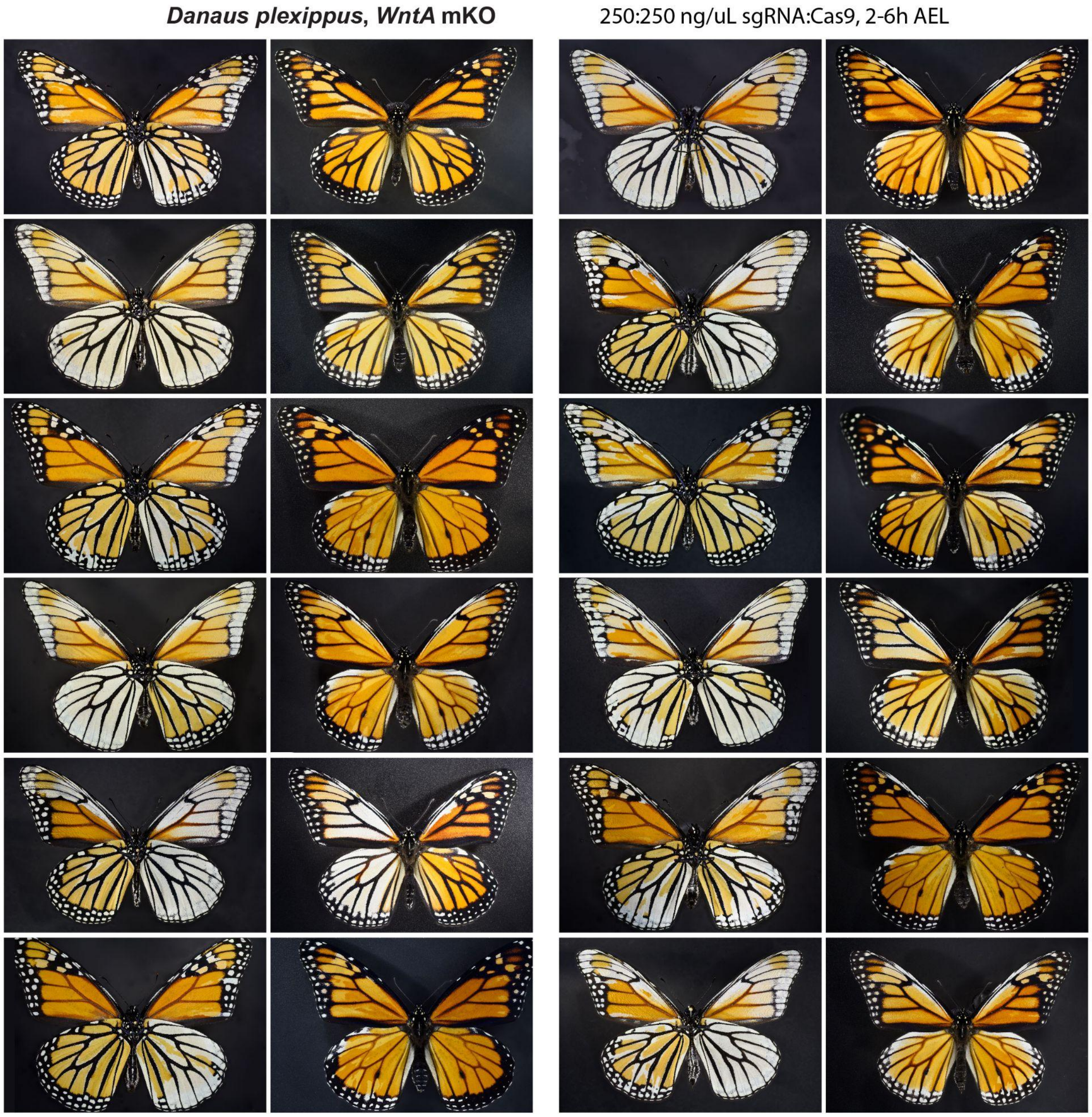

Fig. S2. Representative *D. plexippus WntA* crispant phenotypes. Ventral (left image) juxtaposed to dorsal (right image) sides for each individual.

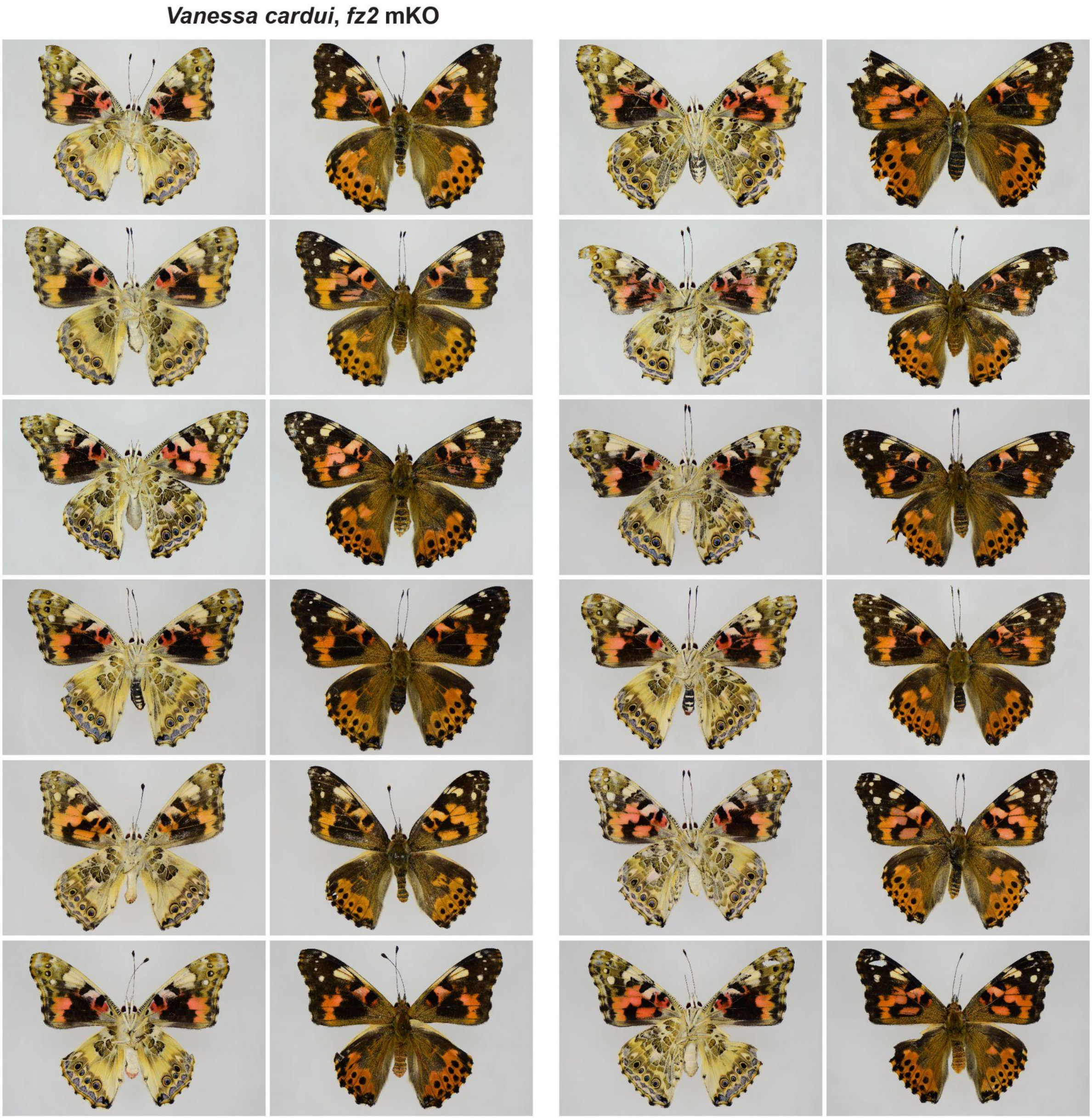

Fig. S3. Representative *V. cardui fz2* crispant phenotypes. Ventral (left image) juxtaposed to dorsal (right image) sides for each individual.

Development • Supplementary information

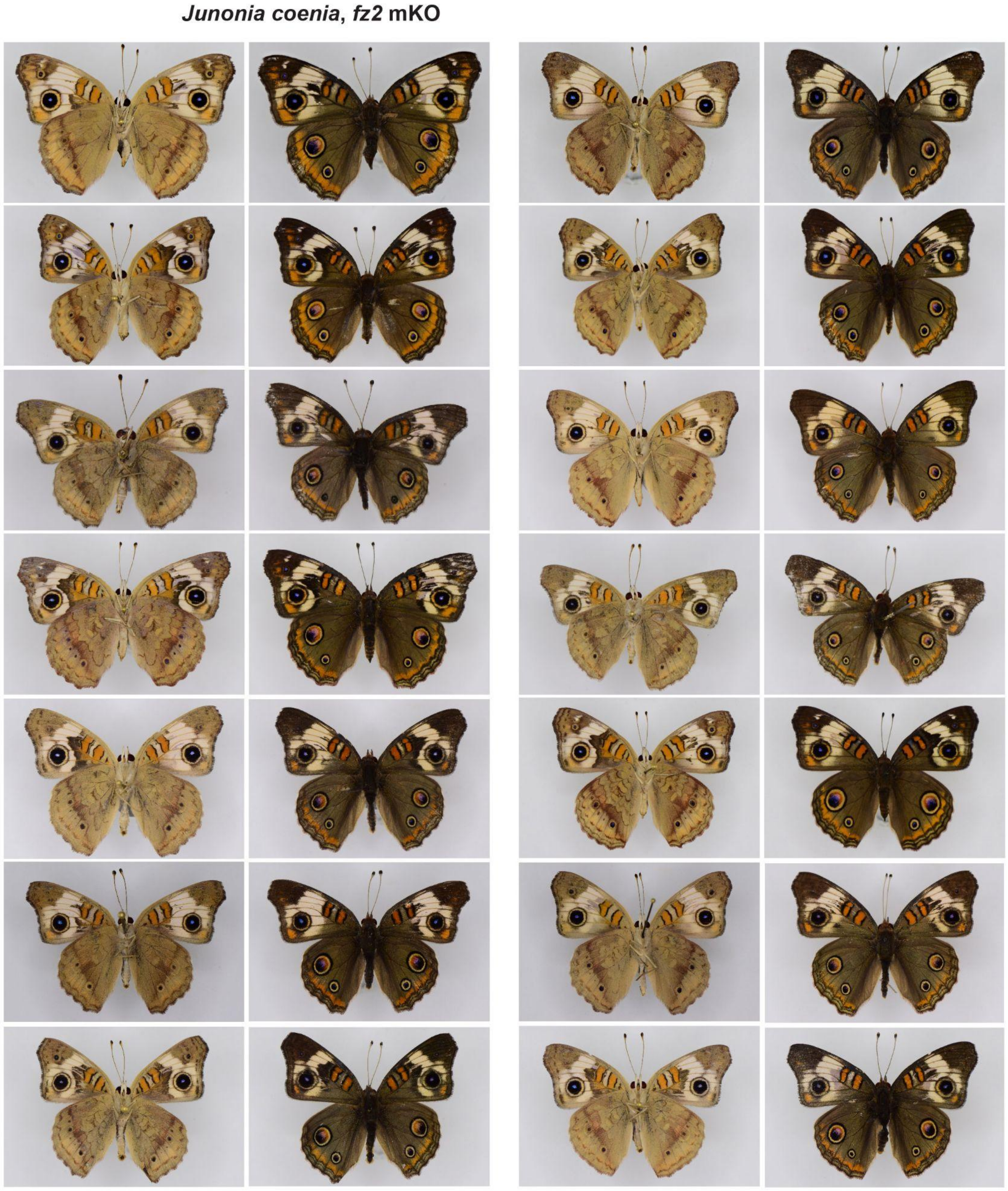

Fig. S4. Representative *J. coenia fz2* crispant phenotypes. Ventral (left image) juxtaposed to dorsal (right image) sides for each individual.

Development • Supplementary information

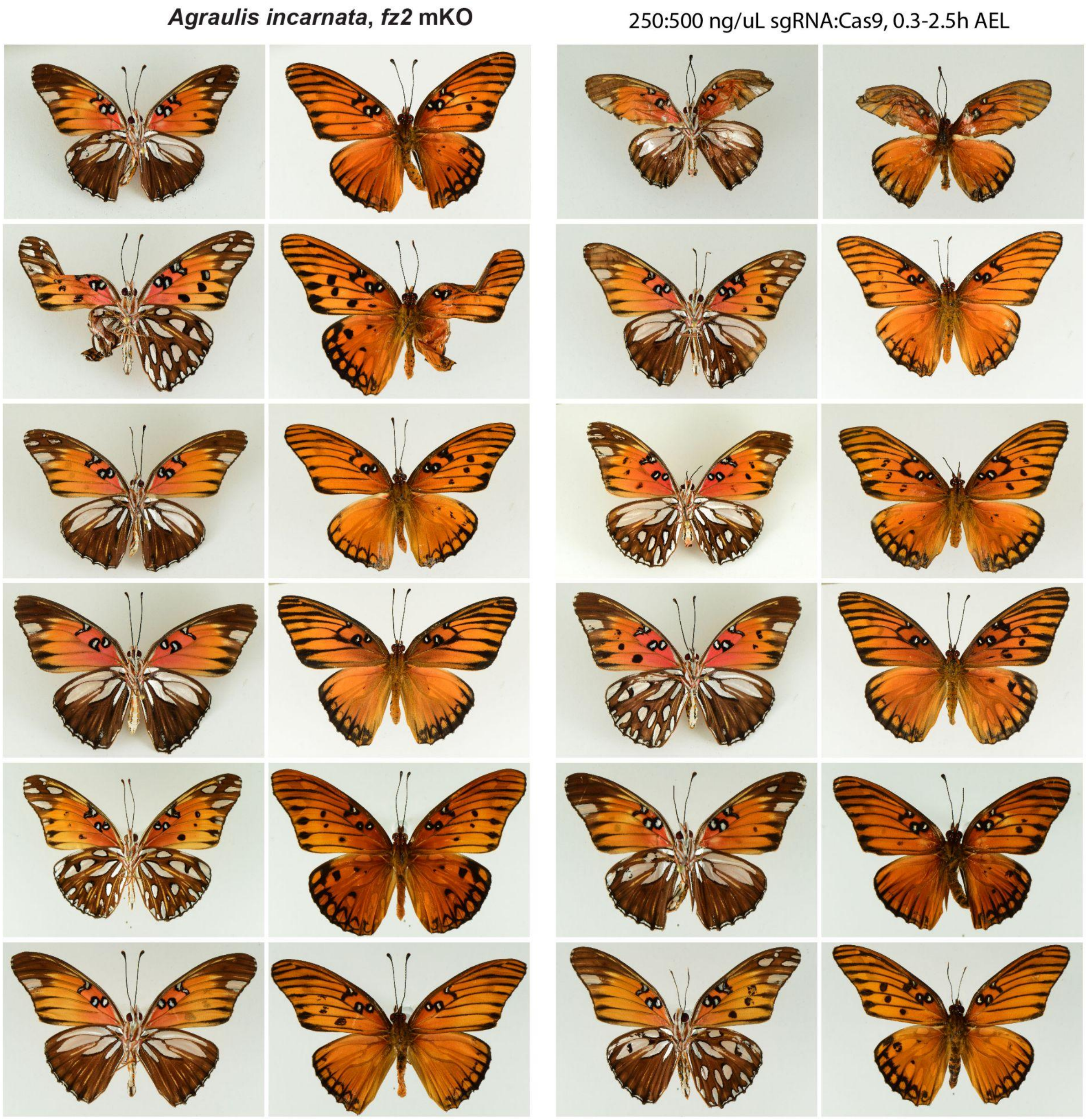

Fig. S5. Representative *A. incarnata fz2* crispant phenotypes. Ventral (left image) juxtaposed to dorsal (right image) sides for each individual.

Development • Supplementary information

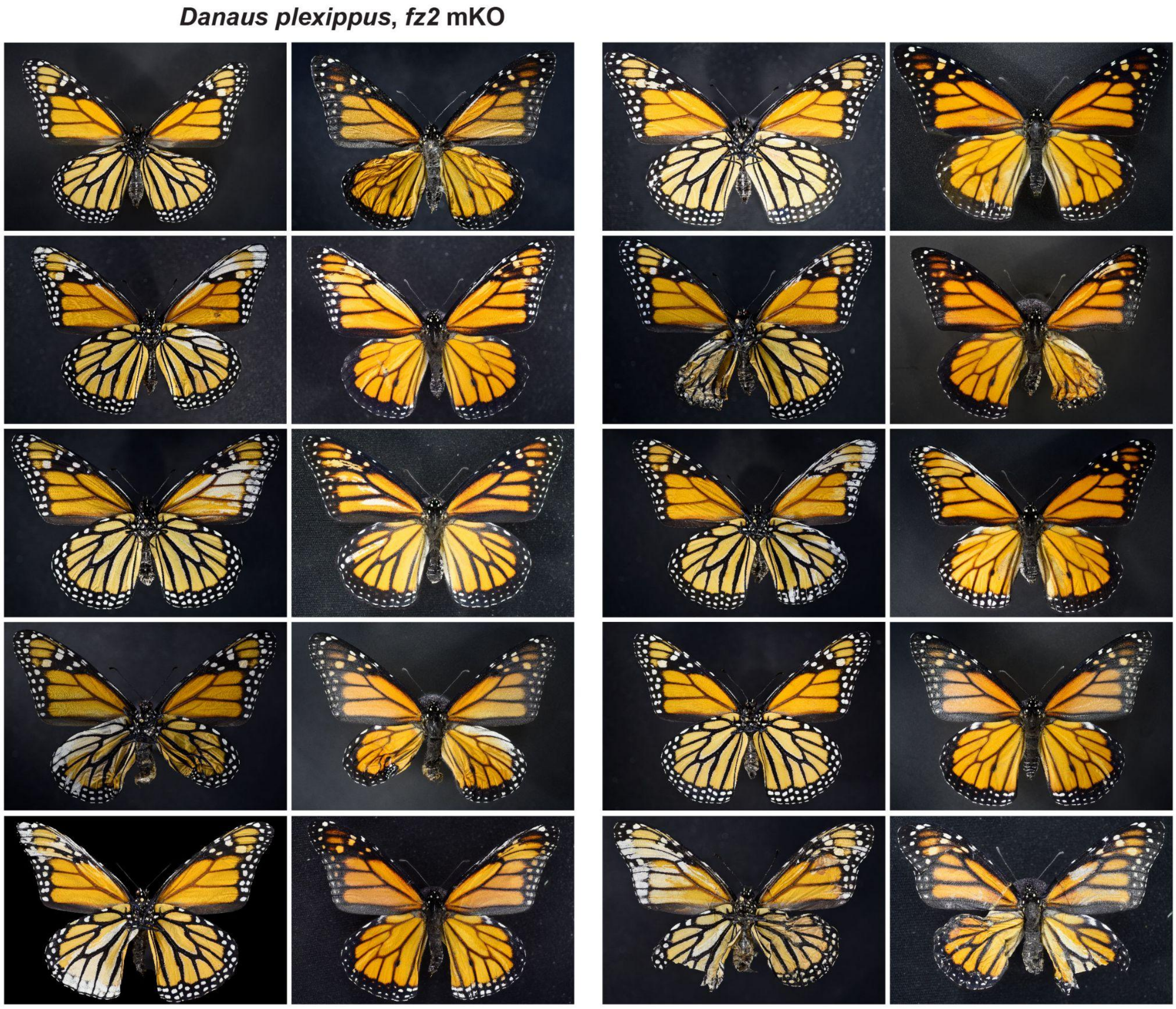

Fig. S6. Representative *D. plexippus fz2* crispant phenotypes. Ventral (left image) juxtaposed to dorsal (right image) sides for each individual.

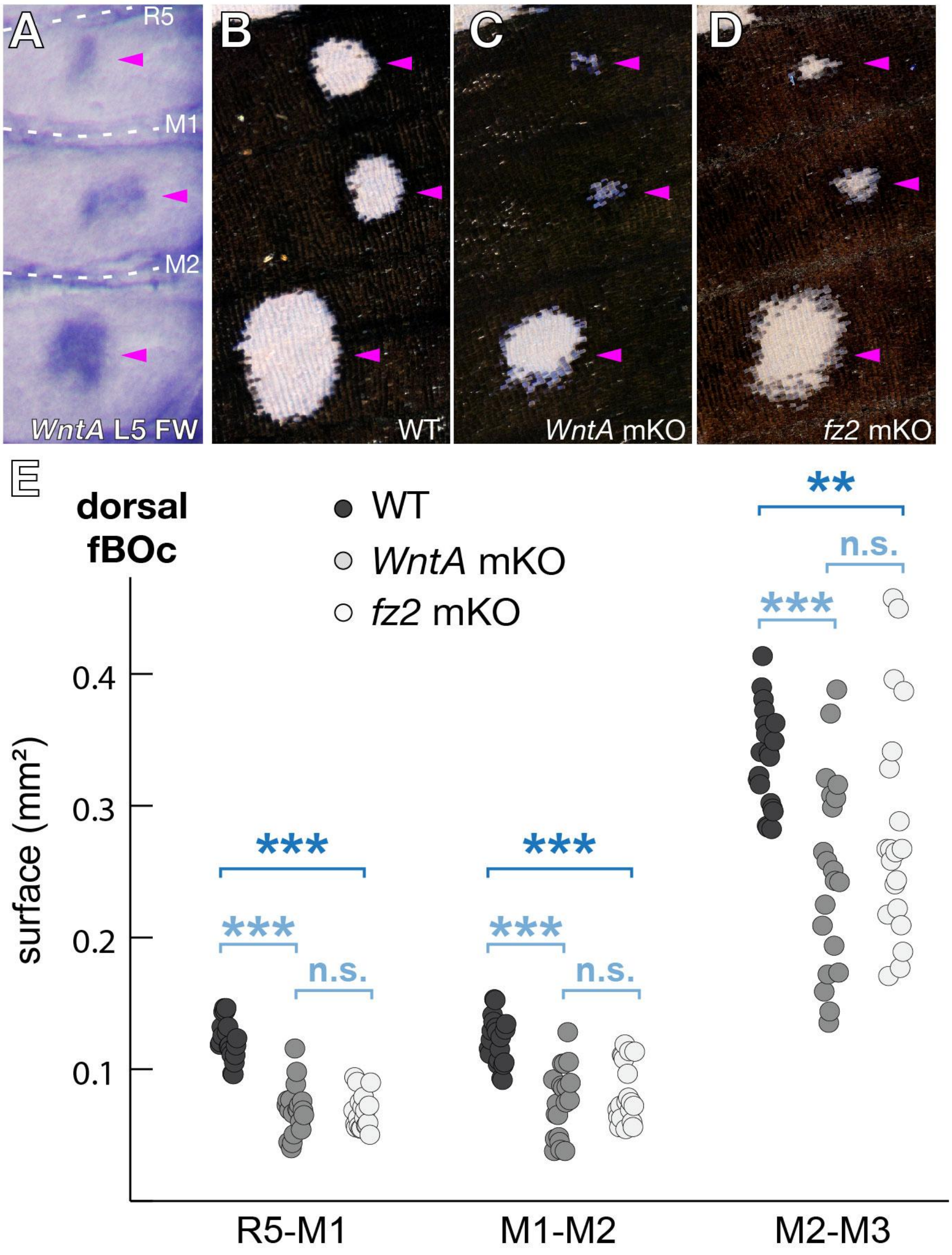

Fig. S7. *fz2* mKOs phenocopy *WntA*-deficient reductions in the forewing Border Ocelli (fBOC) of *V. cardui.* (A) *In situ* hybridization showing *WntA* expression in the presumptive fBOc of *V. cardui* (arrowheads), situated between the R_5-M_3 veins, in late fifth instar wing disks. This fBOc expression persists in pupal stages (**Fig. 2**), and has not been described in nymphalids other than *V. cardui* to date. **(B-D)** Reduction of fBOc compared to WT in both *WntA* and *fz2* crispants, here in dorsal views. **(E)** Surface measurements of dorsal fBOc reveal significant reduction in both *WntA* and *fz2* mutant contexts (Mann-Whitney U-tests, ** : p <0.01 ; ***: p <0.001 ; n.s. : non-significant). Figure adapted from (Mazo-Vargas et al., 2017) with *fz2* data added.

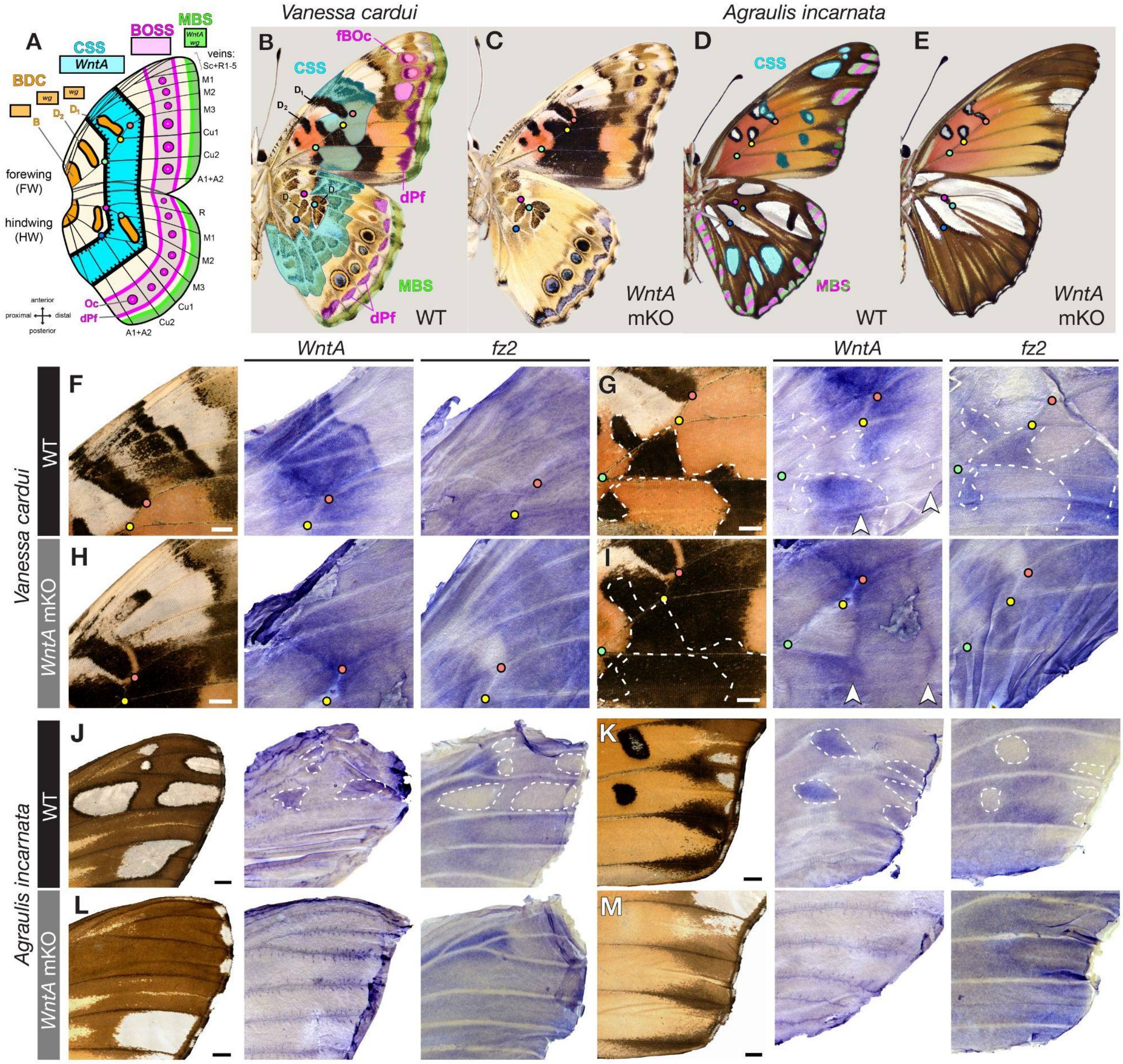

Fig. S8. Expression of *WntA* and *fz2* in pupal wings is under the control of positive and negative feedback. (A) The Nymphalid groundplan (NGP) consists of four symmetry systems : Baso-Discal Complex (BDC, orange), Central Symmetry System (CSS, cyan), Border Ocelli Symmetry System (BoSS, magenta), and Marginal Band System (MBS, green). Color dots mark vein intersection landmarks (red: crossvein-M_3; yellow: M_3-Cu_1; green: Cu_1-Cu_2). **(B)** Derivation of the NGP in ventral *V. cardui*. fBOc : forewing Border Ocelli; dPf : distal Parafocal elements. **(C)** *WntA* loss-of-function impacts the *V. cardui* CSS, fBOc, dPf, and MBS patterns. **(D)** Derivation of the NGP in ventral *A. incarnata*. The CSS is dislocated, and marginal patterns may include partial homology with dPf elements (hashing). **(E)** *WntA* loss-of-function impacts the *A. incarnata* CSS and marginal patterns, and expands the anterior hindwing silver spots. **(F-I)** ISH for *WntA* and *fz2* mRNA across WT and *WntA* crispant wings at 13-17% pupal development (N = 3-4 replicates per experiment). In *WntA* mKO, *WntA* is modified (*e.g.* arrowheads), and *fz2* is de-repressed and becomes ubiquitous. (**J, L**) *A. incarnata* forewing anterior CSS. (**K, M**) *A. incarnata* posterior forewing and margin featuring the two CSS silver spots that flank the Cu_1 vein. Contralateral *WntA-fz2* stains were obtained for F-I and K. Scale bars = 1 mm.

Development • Supplementary information

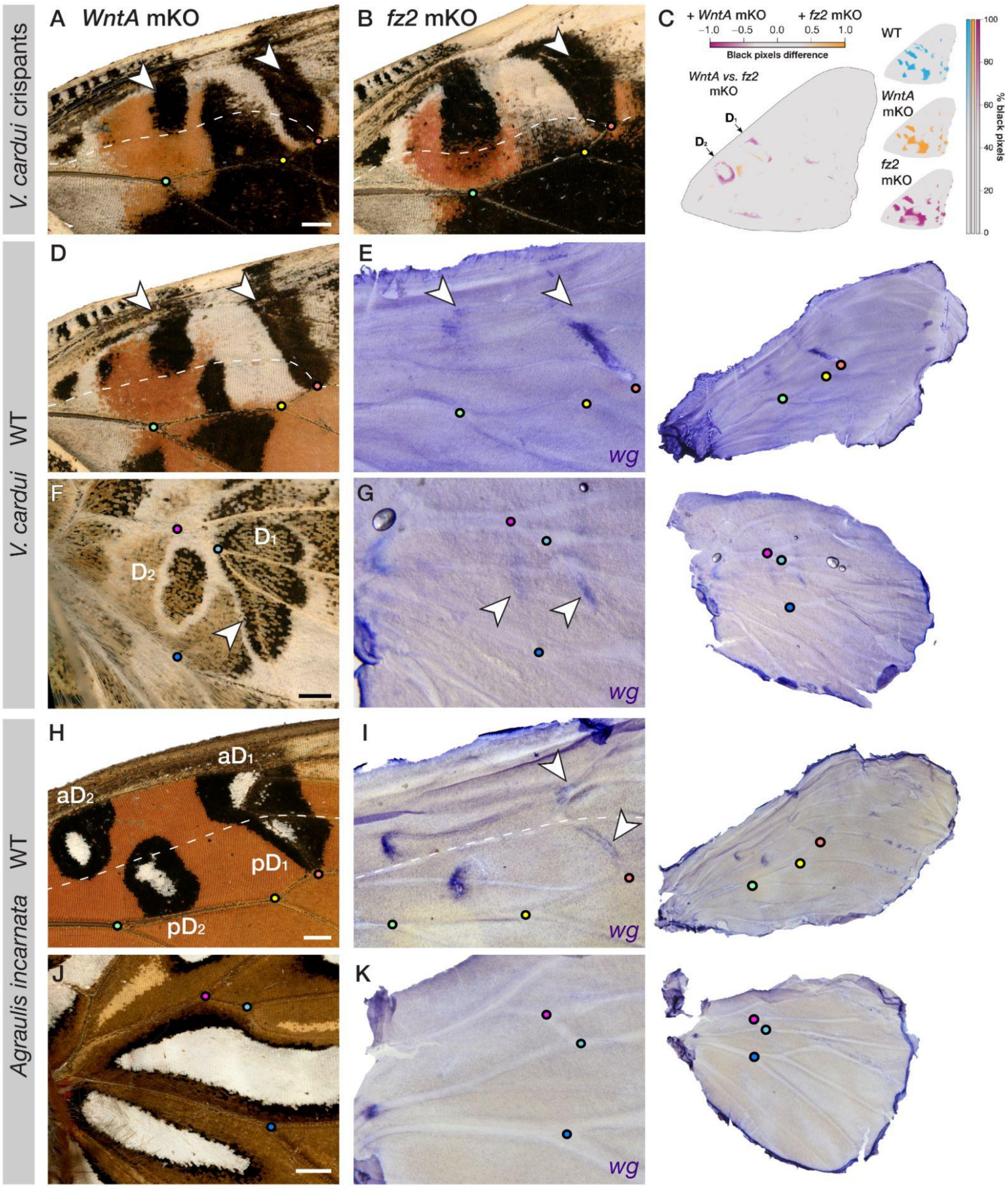

Fig. S9. Size regulation of Discalis elements may involve Wg/Fz2 signaling in ventral *V. cardui* and *A. incarnata* forewings. (A) The D_1 and anterior D_2 (aD_2) elements are unaffected by *WntA* mKOs. **(B)** D_1 and aD_2 show an expansion of black in *fz2* KOs. **(C)** *R/patternize* heatmap comparing *WntA* vs. *fz2* mKO forewings. Black scale expansions were consistently observed in D_1 and D_2 across all the *fz2* mKO samples (arrows). The upper row features black pixel distributions across WT (N = 20 images), *WntA* mKO (N= 20), and *fz2* mKO (N= 40). **(D)** Pupal forewing expression of *wg* marks presumptive aD_2 and D_1 patterns, suggesting exaggerated *fz2* phenotypes may be due to a role of Wg/ Fz2 signaling in inhibiting their size. **(E)** Pupal hindwing expression of *wg* marks the Basalis (B), D_2, and D_1 elements. These three elements remain after *WntA* and *fz2* loss-of-function. **(F)** Pupal forewing of *A. incarnata* is marked by *wg* in the presumptive D_2 and D_1 patterns similar to in *V. cardui.* **(G)** Pupal hindwing expression of *wg* is only in the presumptive B element. **(H-K)** In *A. incarnata* 15-17% pupal wings, *wg* is most prominent in the forewing D_2 (split across the anterior and posterior compartments, here marked with the M_2 vein as dotted line), weakly expressed in the forewing D_1, and absent from hindwings where D elements are missing. Scale bars = 1 mm

Development • Supplementary information

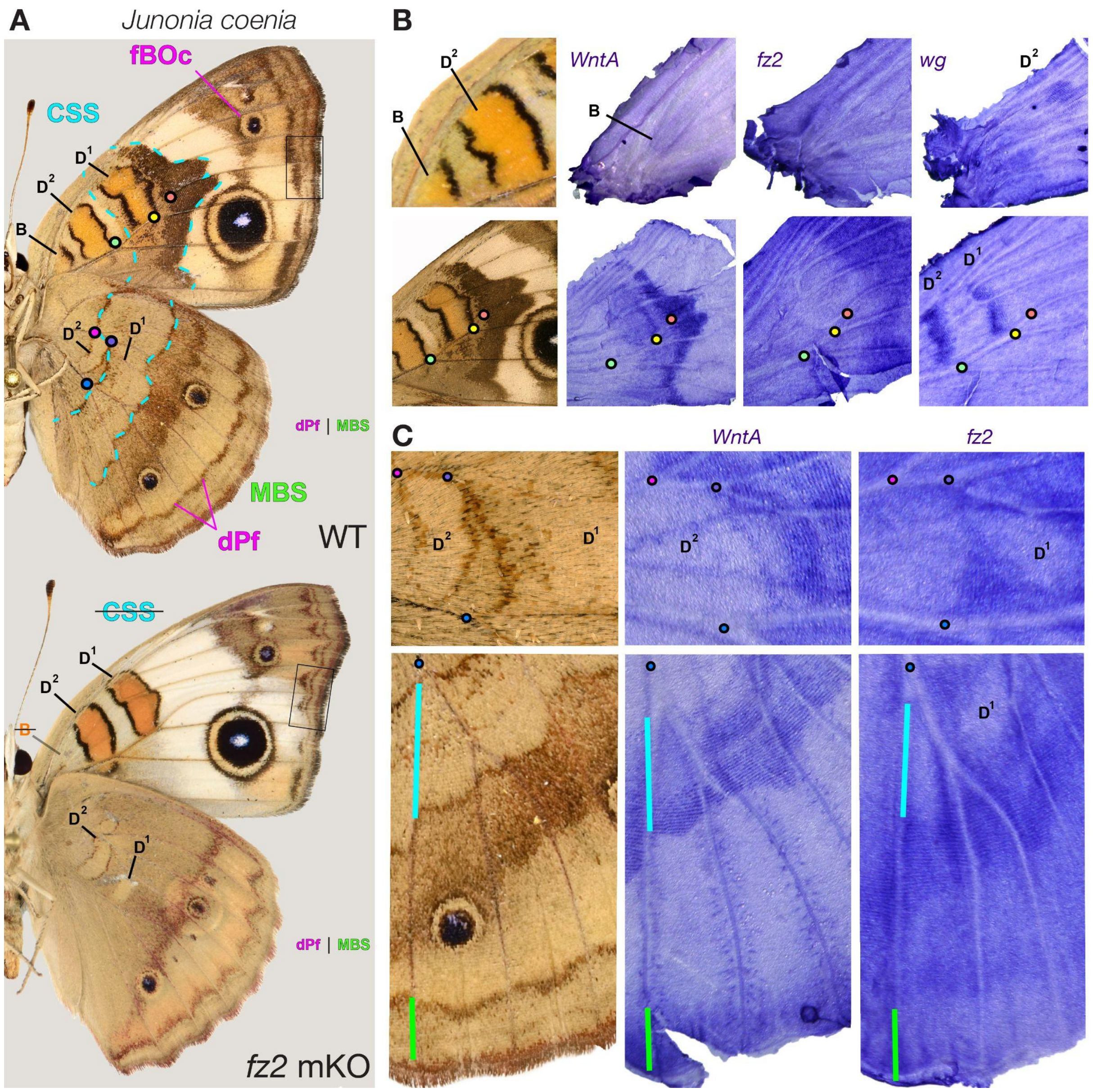

Fig. S10. Conserved functions of *WntA* and *fz2* in a third nymphalid butterfly. (A) *J. coenia* wild type with annotated NGP elements and *fz2* mKO mutant. *fz2* crispants show a complete loss of the Basalis (B) and CSS elements, as well as a distalization and reshaping of the dPf elements, most visible in the forewings (box). **(B)** ISH of *WntA*, *fz2* and *wg* in the proximal regions of the *J. coenia* WT forewing at 17% pupal development (n = 5 replicates). *WntA* induces the B and CSS elements, with visible *fz2* depletion at this stage, consistently with a negative feedback of WntA/Fz2 signaling on *fz2*; *wg* is strongly expressed in D_1 and D_2. **(C)** Expression of *WntA* and depletion of *fz2* in the hindwing CSS (cyan) and MBS region (green) is consistent with a role of the WntA/Fz2 signaling on inducing the CSS and positioning peripheral patterns.

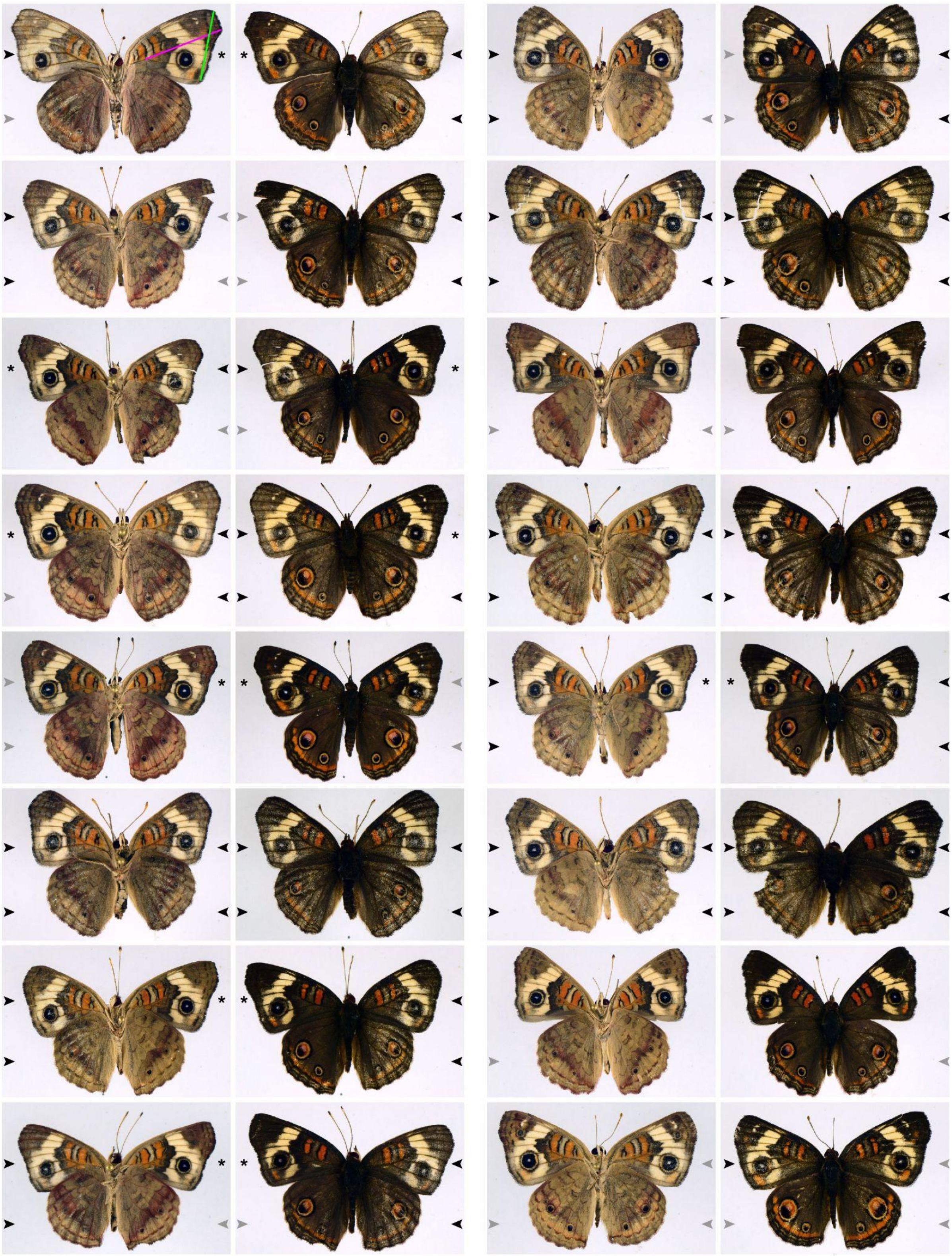

Fig. S11. Mosaic *fz1* crispants show wings with disorganized scale arrays, short forewings, and no pattern defects in *J. coenia*. PCP phenotypes are visible in these whole-specimen views as zones of apparent wing wear. Left: ventral side ; right : dorsal side. Black arrowheads : extensive PCP phenotypes ; gray arrowheads : wing surface with small PCP clones ; no arrowhead : surface with WT phenotype. Asterisks denote forewings that are WT on ventral and dorsal sides, and contralateral to a PCP-mutant forewing. Wing length (magenta) and width (green) between vein landmarks were measured in these PCP-asymmetric forewing pairs (N=7). Mutant wings are significantly shorter than WT in length (Wilcoxon signed rank test, p = 0.039) but not in width (p = 1).

Development • Supplementary information

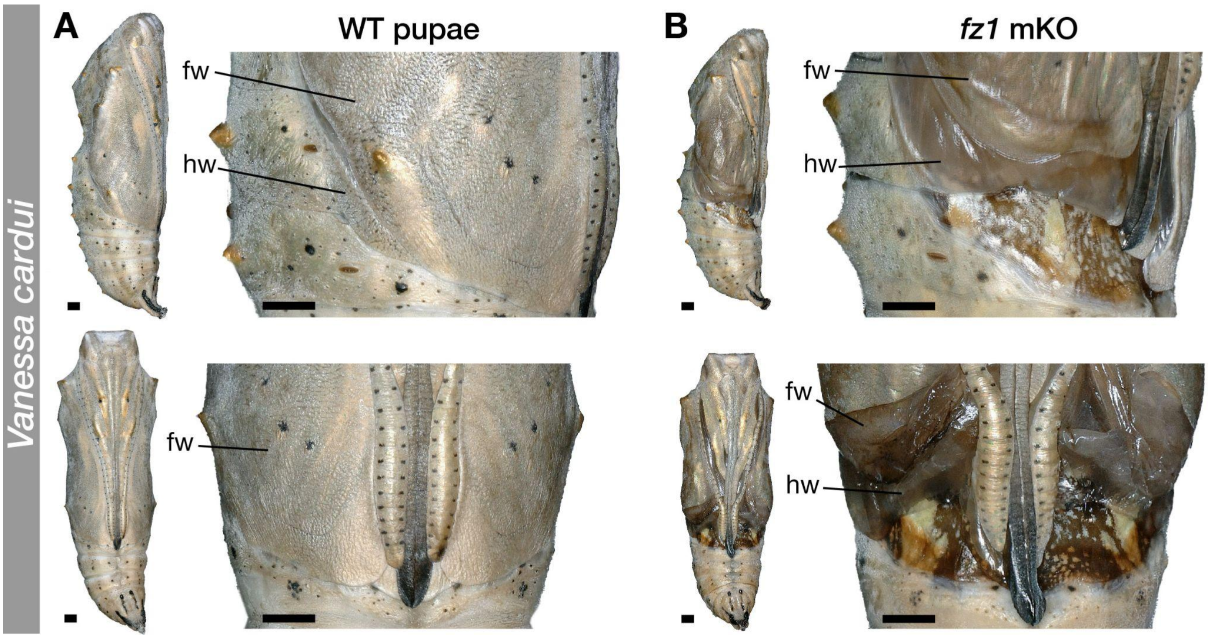

Fig. S12. Pupal wing defects with incomplete wing growth and cuticularization in *fz1* crispants of *V. cardui*. (A) WT pupa in lateral (top) and ventral (bottom) views, with insets showing the distal portion of the pupal forewing (fw) and hindwing (hw) suturing with thoracic and abdominal cuticle. (**B**) Two examples of pupal phenotypes observed in *V. cardui fz1* crispants (N=13), with incomplete wing growth and distal sealing. Antennae, proboscis and legs, which bundle around the ventral midline, appeared unaffected. Scale bars = 1 mm.

Development • Supplementary information

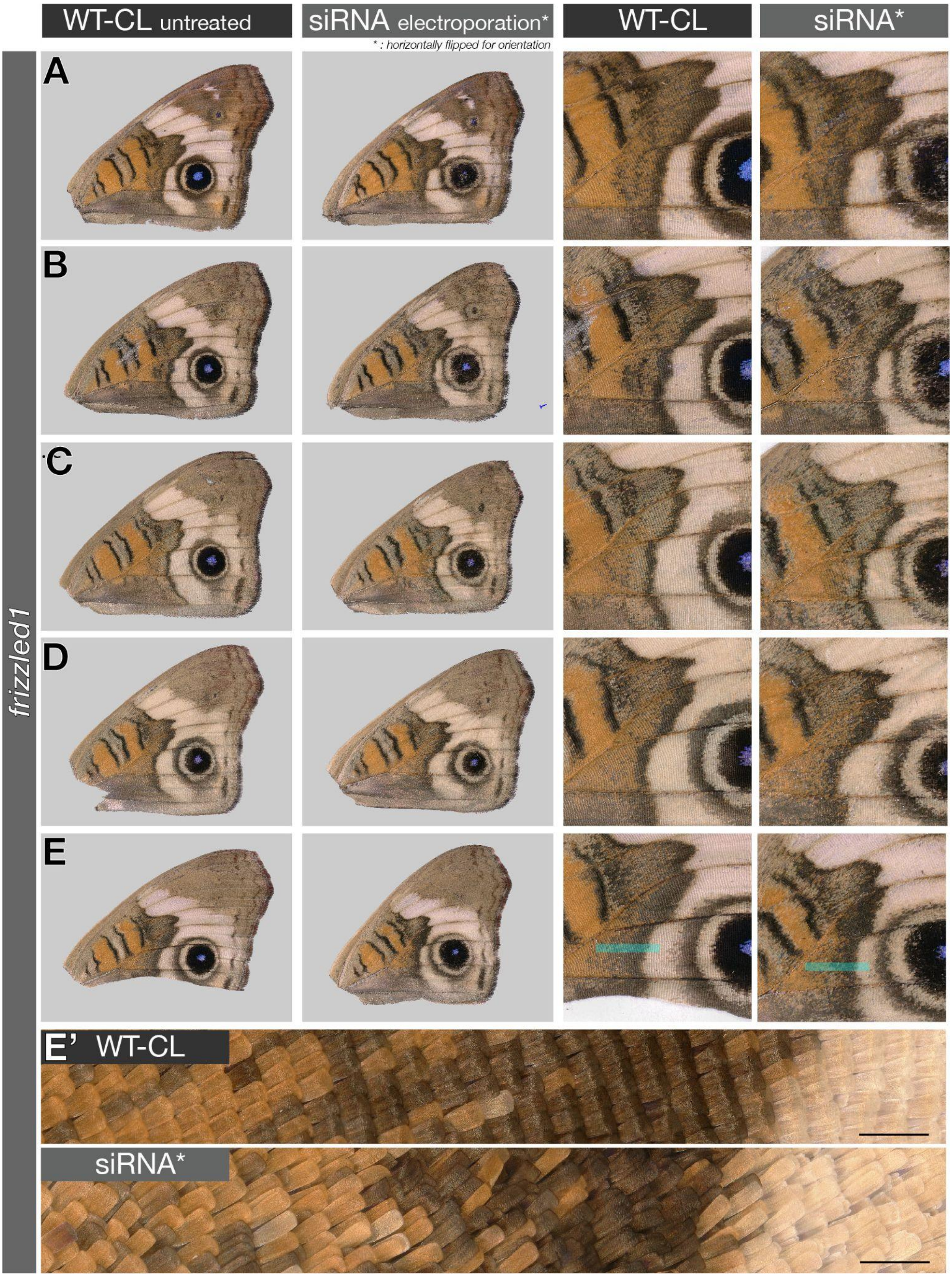

Fig. S13. Pupal RNAi electroporation knockdowns of *fz1* in *J. coenia*. Ventral views of single specimens electroporated with *fz1* DsiRNA on the ventral right forewing, shown here next to their contralateral wild-type control (WT-CL). PCP-like phenotypes are visible as fields of straightened scales (lacking normal curvature) and lacking regular organization under high magnification in all five replicates (as shown in E', bottom panel) and were not observed in controls. PCP-like phenotypes were pronounced in *A. incarnata* knockdown experiments (**Fig. 4G-J**). Scale bars: E' = 200 μm.

Development • Supplementary information

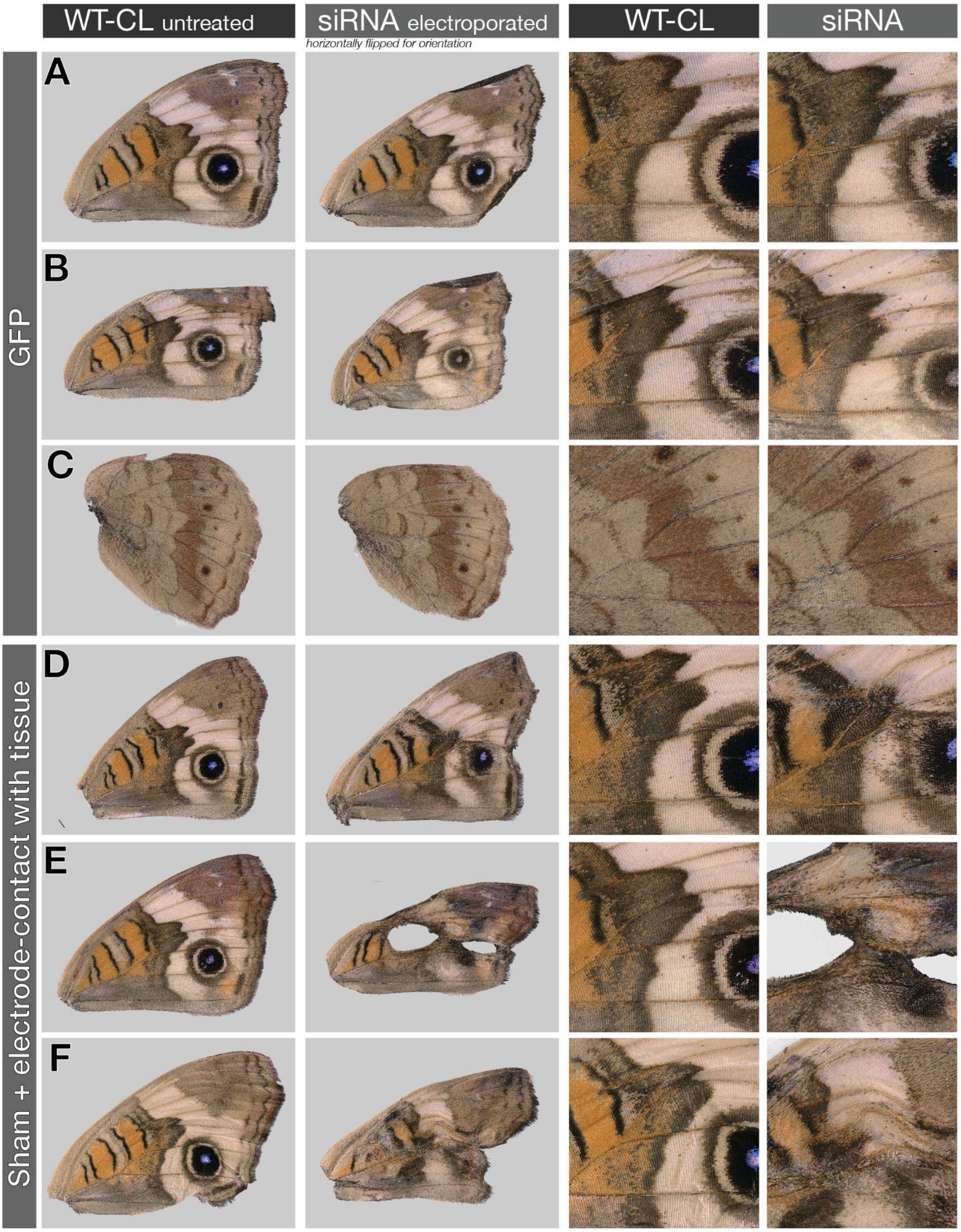

Fig. S14. Pupal RNAi electroporation sham-negative controls and description of stress artefacts in *J. coenia*. Examples of artefacts observed in sham electroporation procedures with GFP DsiRNA (A-B, ventral forewings ; C, ventral hindwing), and following electroporation with the electrode directly in contact with the wing tissue (D-F). See Methods section for details.

Development • Supplementary information

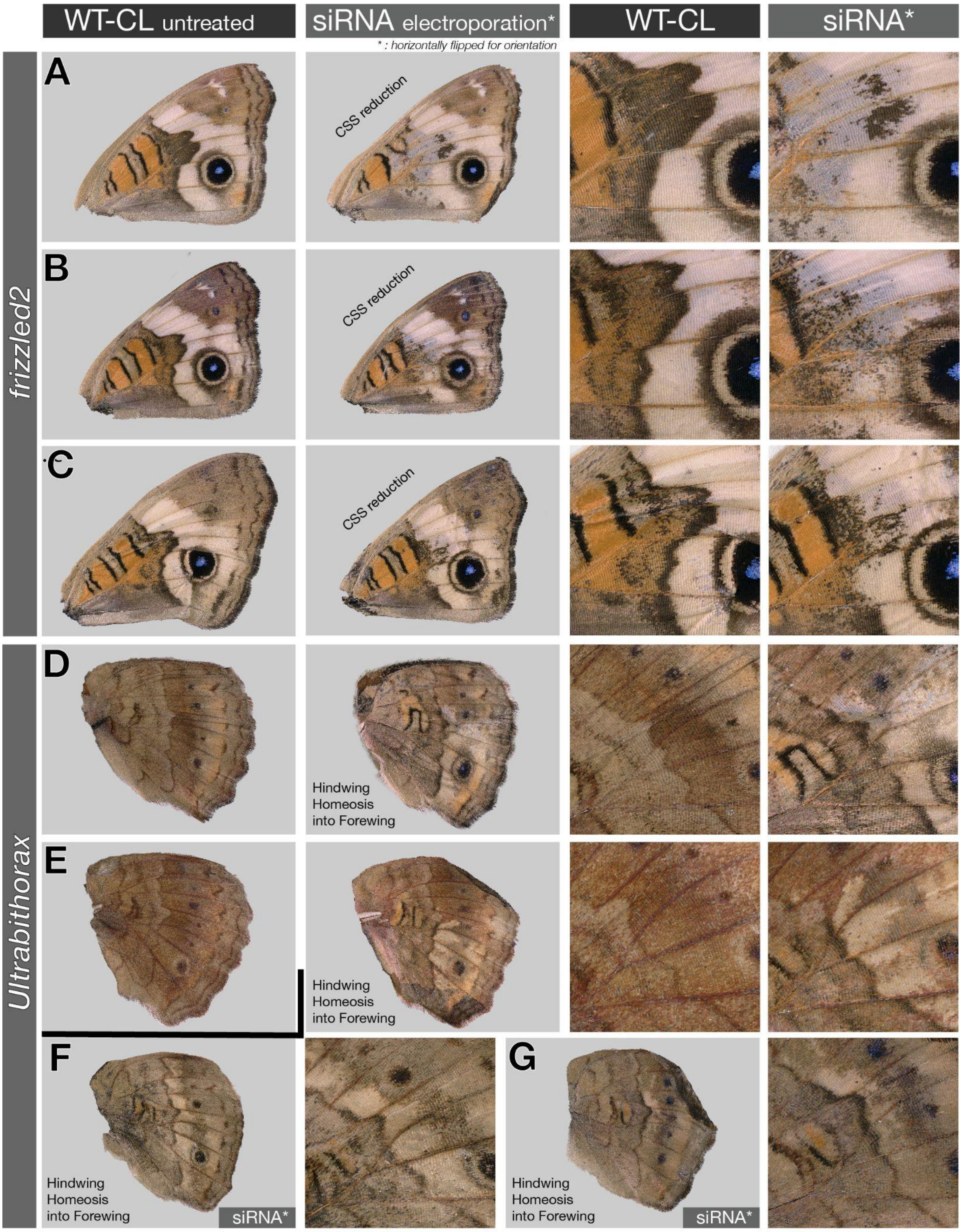

Fig. S15. Pupal RNAi electroporation knockdowns of positive controls *fz2* and *Ubx* in *J. coenia*. Representative examples of knockdown phenotypes obtained in forewings with *fz2* DsiRNA (A-C) and in hindwings with *Ubx* DsiRNA (D-G). Effects on CSS reduction (*fz2*) and partial homeoses of hindwings into forewings (*Ubx*) are consistent with expectations from CRISPR-induced KO phenotypes and validate our implementation of the RNAi electroporation method.

Development • Supplementary information

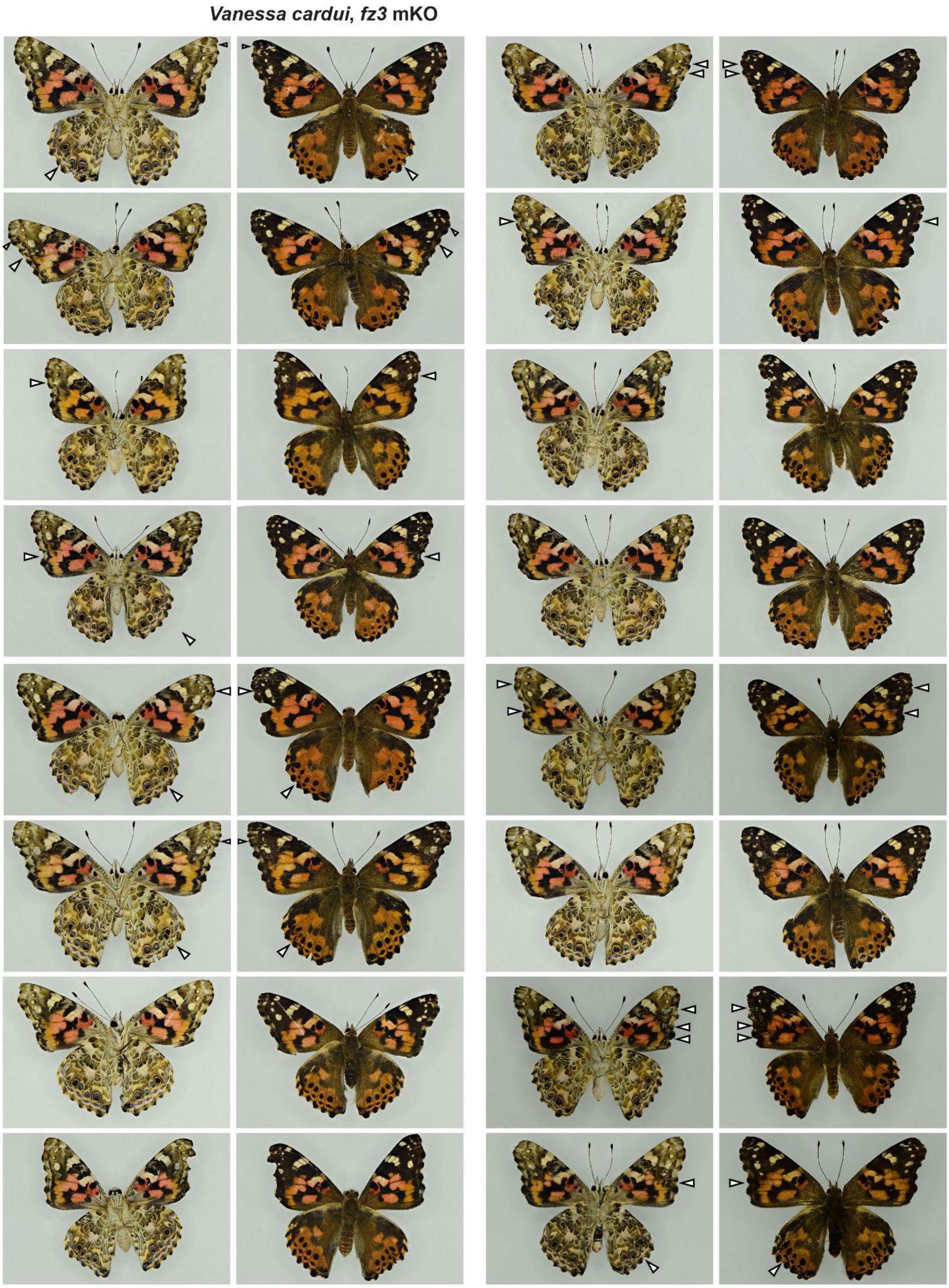

Fig. S16. Representative *V. cardui fz3* crispant phenotypes. Ventral (left image) juxtaposed to dorsal (right image) sides for each individual. Arrowheads indicate ectopic veins.

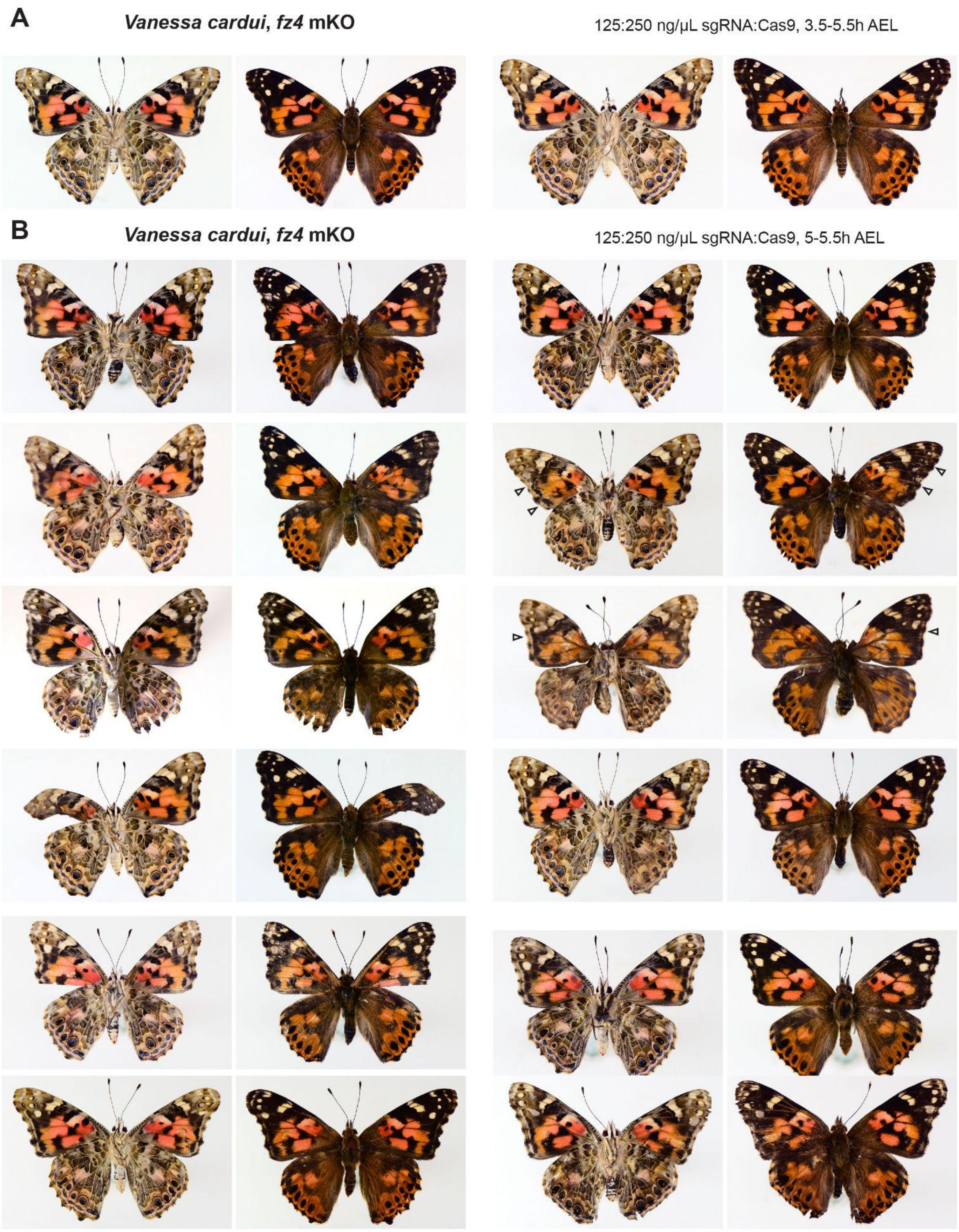

Fig. S17. Representative *V. cardui fz4* crispant phenotypes. Ventral (left image) juxtaposed to dorsal (right image) sides for each individual.

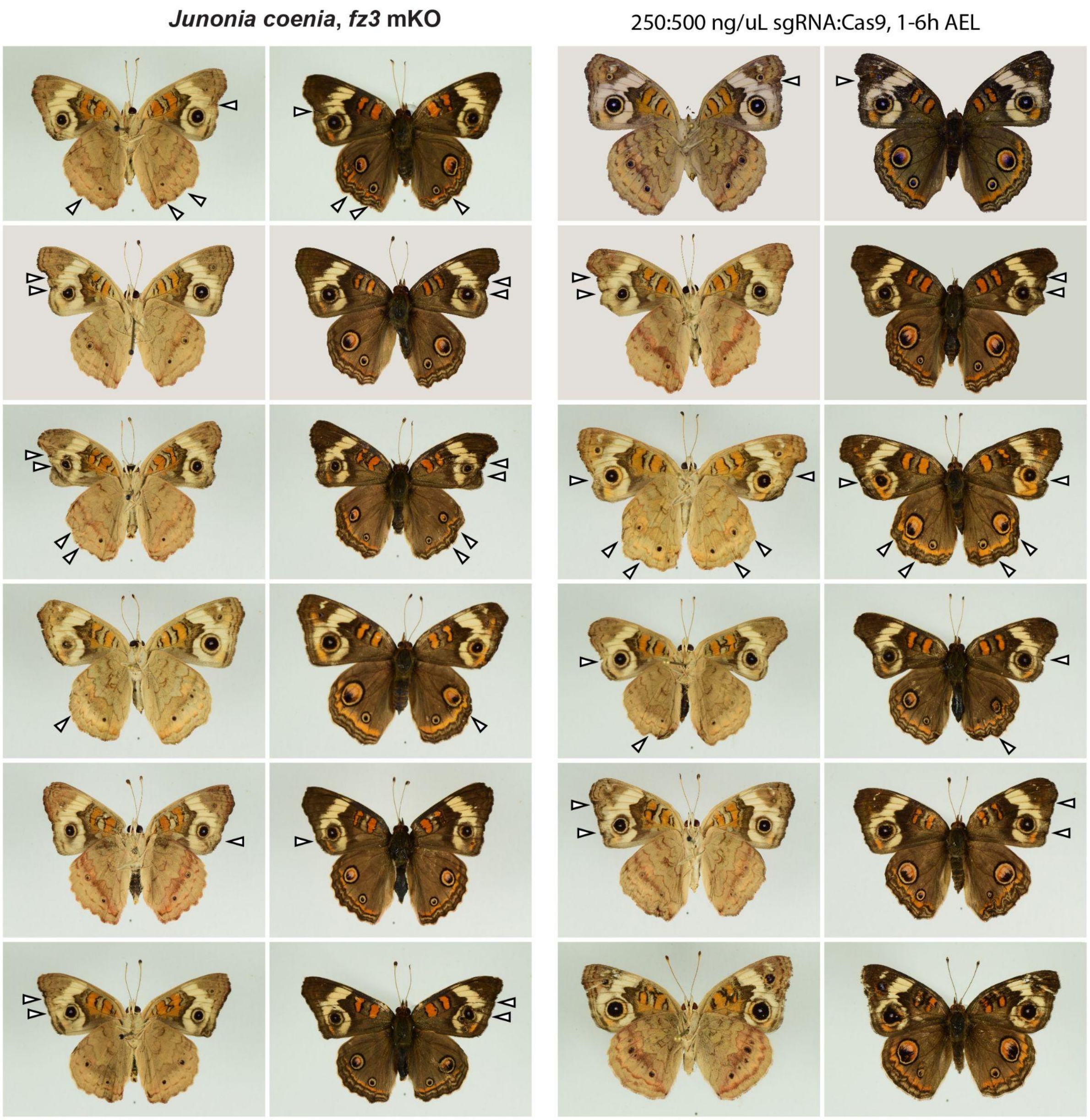

Fig. S18. Representative *J. coenia fz3* crispant phenotypes. Ventral (left image) juxtaposed to dorsal (right image) sides for each individual. Arrowheads indicate ectopic veins.

Development • Supplementary information

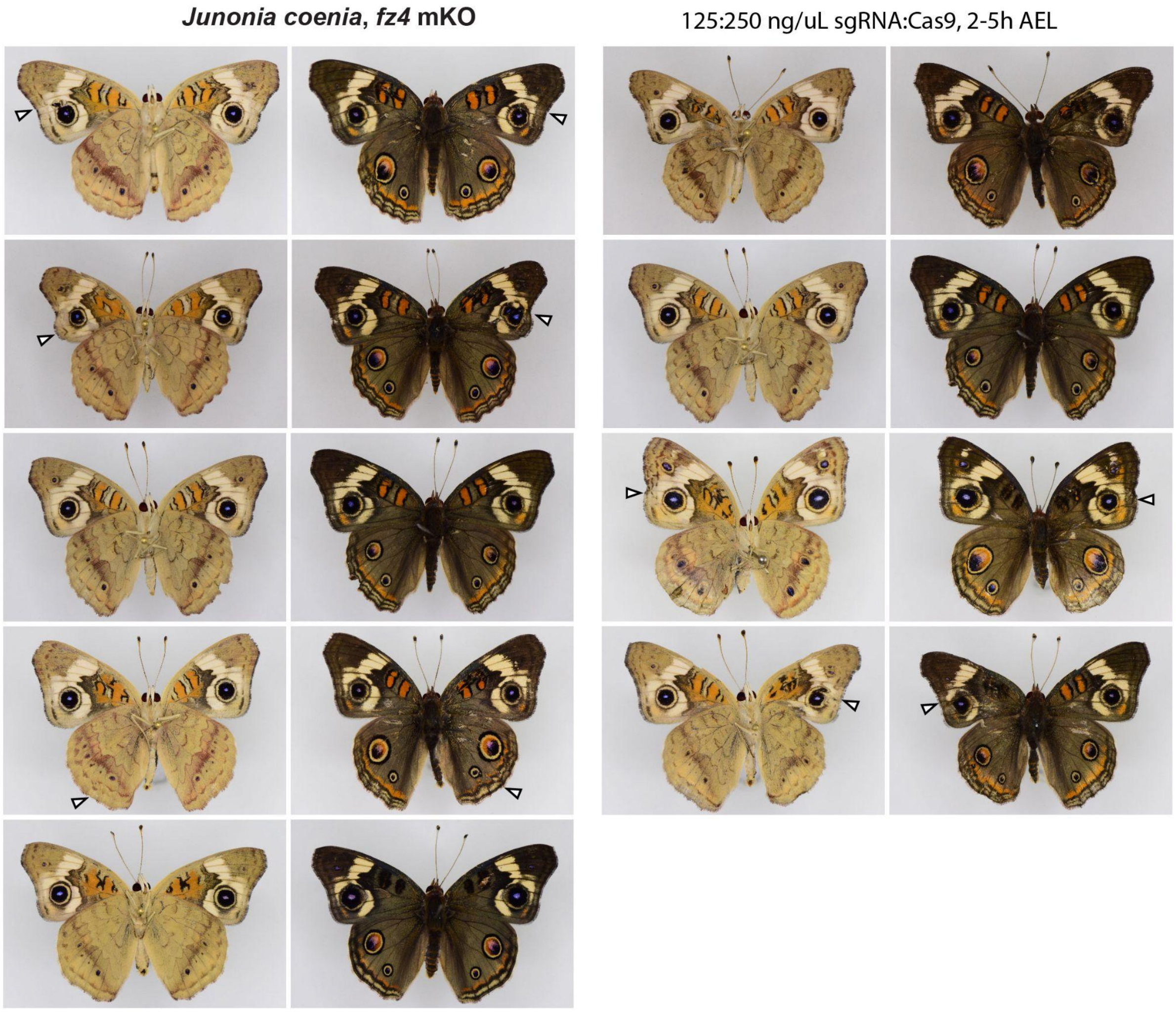

Fig. S19. Representative *J. coenia fz4* crispant phenotypes. Ventral (left image) juxtaposed to dorsal (right image) sides for each individual.

Development • Supplementary information

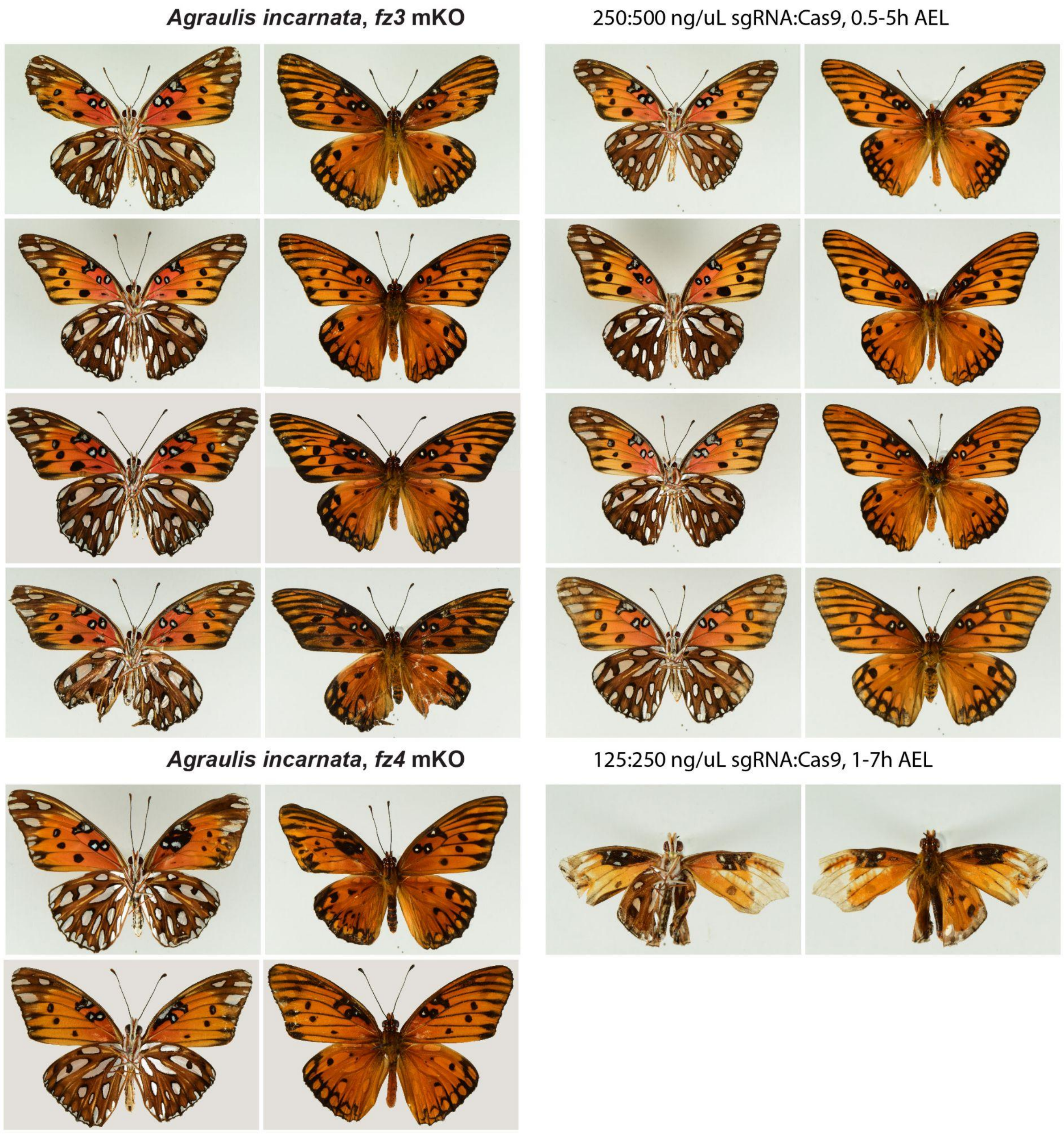

Fig. S20. Representative *A. incarnata fz3* and *fz4* crispant phenotypes. Ventral (left image) juxtaposed to dorsal (right image) sides for each individual

Development • Supplementary information

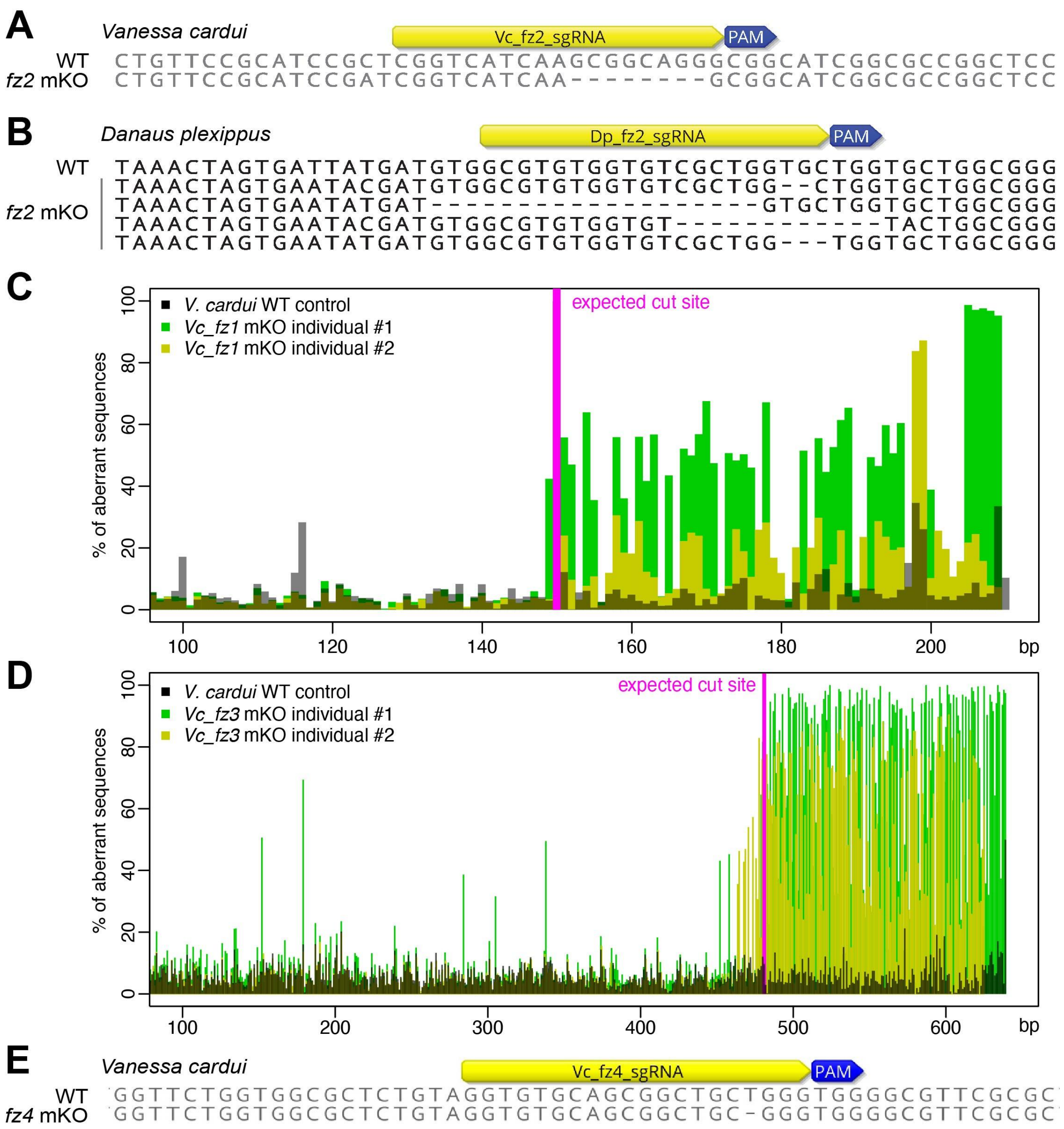

Fig. S21. Genotyping of G_0 crispants. (A) Detection of a single frameshift *fz2* mutation clone in a *V. cardui* crispant by direct Sanger sequencing. No wildtype sequence was recovered in this individual, implying the existence of a second mutant allele that was not amplified by PCR. DNA was directly amplified with the Phire Tissue Direct PCR Master Mix. **(B)** Detection of multiple frameshift mutations in two *D. plexippus fz2* crispants following colony PCR. **(C-D)** Detection ofindels at the predicted sgRNA cutting sites in *V. cardui fz1* and *fz3* crispants using sequence trace decomposition with the TIDE tool (Brinkman et al., 2014). Aberrant sequencing signals after the predicted cut site are due to the presence of indel alleles, resulting in mixed chromatograms. **(E)** Detection of a single frameshift *fz4* mutation clone in a *V. cardui* crispant by direct Sanger sequencing.

Development • Supplementary information

Table S1. Summary of CRISPR-target mutagenesis experiments (continued on next page) n.r. : not recorded

CRISPR target	Target Species	sgRNA	Injection time hrs AEL	[Cas9: sgRNA] ng/µL	Embryos Ninj	Larvae L1, Nlar	Hatching rate Nlar/Ninj	Adults Nadu	Survival rate Nadu/Ninj	Adult (pupal) crispants	Wing phenotypes
fz1	*V. cardui*	*Vc+Jc_fz1*	3-4	250 : 125	136	102	75%	29	21%	0 (5)	Growth defects
			3-6	250 : 125	220	171	78%	8	4%	0 (9)	Growth defects
	J. coenia	*Vc+Jc_fz1*	2-7	500 : 250	422	n.r.	n.r.	42	10%	8	PCP-like, mild growth defects
			1-5	250 : 125	1080	n.r.	n.r.	71	7%	9	PCP-like, mild growth defects
			4.5-8	500 : 250	546	259	47%	78	14%	2	PCP-like, mild growth defects
		Jc_fz1	2-8	250 : 125	2346	100	4%	46	2%	0	-
			0.5-6	125 : 62.5	879	183	21%	49	6%	14	PCP-like, mild growth defects
fz2	*V. cardui*	*Vc_fz2*	2-5	500 : 250	378	48	5%	18	5%	9	Phenocopy of *WntA* KO
			2.5-6	250 : 125	604	213	56%	29	5%	14	Phenocopy of *WntA* KO
			3.5-5.5	250 : 125	642	238	39%	42	7%	26	Phenocopy of *WntA* KO
	J. coenia	*Jc_fz2*	1-5	500 : 250	1230	522	42%	187	15%	35	Phenocopy of *WntA* KO
		Ai+Jc_fz2	2-3.5	500 : 250	515	n.r.	n.r.	56	11%	44	Phenocopy of *WntA* KO
			5-6	250 : 125	192	>185	>96%	185	27%	51	Phenocopy of *WntA* KO
	A. incarnata	*Ai+Hm_fz2*	0.3-2.5	500 : 250	80	34	40%	20	24%	19	Phenocopy of *WntA* KO
	H. melpomene	*Ai+Hm_fz2*	0.5-4	500 : 250	124	21	17%	10	10%	2	Phenocopy of *WntA* KO
			0.5-3.5	500 : 250	104	30	29%	8	8%	0	-
	H. erato		0.5-1.5	500 : 250	46	14	30%	7	15%	1	Phenocopy of *WntA* KO
	D. plexippus	*Dp_fz2*	3-4	250 : 250	35	30	86%	12	34%	7	Phenocopy of *WntA* KO
			4-6	250 : 250	25	22	88%	16	64%	9	Phenocopy of *WntA* KO

Development • Supplementary information

CRISPR target	Target Species	sgRNA	Injection time hrs AEL	[Cas9: sgRNA] ng/µL	Embryos Ninj	Larvae L1, Nlar	Hatching rate Nlar/Ninj	Adults Nadu	Survival rate Nadu/Ninj	Adult (pupal) crispants	Wing phenotypes
fz3	*V. cardui*	*Vc_fz3*	1.5-2.5	500 : 250	172	16	9%	3	2%	2	Ectopic veins, Margin perturbation
			2-3.5	500 : 250	230	124	53%	9	4%	0	-
			2.5-4.5	500 : 250	243	132	54%	9	4%	2	Ectopic veins, Margin perturbation
			3-4.5	500 : 250	702	273	39%	44	16%	28	Ectopic veins, Margin perturbation
	J. coenia	*Jc_fz3*	1-6	500 : 250	785	n.r.	n.r.	128	4%	35	Ectopic veins, Margin perturbation
	A. incarnata	*Ai_fz3*	0.5-5	500 : 250	60	41	68%	29	48%	6	Ectopic veins, Margin perturbation
			0.5-5	500 : 250	13	11	85%	8	62%	1	Ectopic veins, Margin perturbation
fz4	*V. cardui*	*Vc_fz4*	3.5-6.5	250 : 125	338	162	48%	24	7%	16	Ectopic veins, D1-2 expansion, Peripheral patterns
			3.5-5.5	250 : 125	324	103	32%	29	9%	17	Ectopic veins, D1-2 expansion, Peripheral patterns
			5-5.5	250 : 125	227	96	43%	17	7%	10	Ectopic veins, D1-2 expansion, Peripheral patterns
	J. coenia	*Jc_fz4*	2-5	250 : 125	431	n.r.	n.r.	164	38%	38	Ectopic veins, D1-2 expansion, Peripheral patterns
	A. incarnata	*Ai_fz4*	1-7	250 : 125	282	52	43%	n.r.	n.r.	5	Ectopic veins, D1-2 expansion, Peripheral patterns
WntA	*V. cardui*	*Vc_WntA_ex4*	0.3-1.5	500 : 250	613	438	71%	179	30%	160	CSS pattern loss, dPf / MBS shifts, fBOC reduction
	A. incarnata	*Ai_WntA_ex4*	0.2-1.5	500 : 250	127	89	70%	27	30%	27	Basalis and CSS pattern loss, dPf and MBS shifts
	D. plexippus	*Dp_WntA_1+2*	2-6	250 : 250	120	n.r.	n.r.	n.r.	n.r.	26	Expansion of white patterns

Table S2. sgRNA sequences for CRISPR mosaic knock-outs

CRISPR target gene	Species	sgRNA name	Target sequence 5' to 3' ; PAM not shown
frizzled1	*V. cardui*	*Vc_fz1_sgRNA*	TATGGCTTCAAGTATCTGG
	J. coenia	*Jc_fz1_sgRNA*	TCGCACAAATGGCGACAGGG
frizzled2	*V. cardui*	*Vc_fz2_sgRNA*	CGGTCATCAAGCGGCAGGG
	J. coenia	*Jc_fz2_sgRNA*	GCGCGCACGCTCACGAGCG
	J. coenia	*Ai+Jc_fz2_sgRNA*	GGTGATCAAGCGGCAGGG
	A. incarnata		
	A. incarnata	*Ai+Hm_fz2_sgRNA*	TGAGCGTCATGAGCGTGG
	H. melpomene		
	H. erato		
	D. plexippus	*Dp_fz2_sgRNA*	GCGTGTGGTGTCGCTGGTGC
frizzled3	*V. cardui*	*Vc_fz3_sgRNA*	CAGTCAAGCTTGATACTGGG
	J. coenia	*Jc_fz3_sgRNA*	CAGTCCAGCTTGATACTAGG
	A. incarnata	*Ai_fz3_sgRNA*	CCGACCATCAAACTCGA
frizzled4	*V. cardui*	*Vc_fz4_sgRNA*	GGTGTGCAGCGGCTGCTGGG
	J. coenia	*Jc_fz4_sgRNA*	GATGTGCAGCGGCTGCTGGG
	A. incarnata	*Ai_fz4_sgRNA*	ACGAGCACATGTGCATGG
WntA	*V. cardui*	*Vc_WntA_ex4_sgRNA*	GGCAGCATTGGCCCATGCGG
	A. incarnata	*Ai_WntA_ex4_sgRNA*	GGCTCGGGCGTGCAGTCGTG
	D. plexippus	*Vc_WntA_sgRNA1*	TGCAGAAGGAAGCCTGCCAC
		Vc_WntA_sgRNA2	GCTATCCGACAAGATGGTTC

Table S3. PCR primer sequences for crispant genotyping

PCR genotyping target	Species	Features	Oligonucleotide sequence 5' to 3'
frizzled1	*V. cardui*	**M13F**, forward	**TGTAAAACGACGGCCAGT**AAGATGCTGGCCTTGAGGTC
		forward, internal	GCTGCTGCATATGTAATGGG
		reverse	TCATGCCCCCATTTGAGACC
frizzled2	*V. cardui*	**M13F**, forward	**TGTAAAACGACGGCCAGT**TCGCCCGAGAACCTCAAAAA
		**PIGtail**, reverse	**GTGTCTT**GTACAGCACGCTGAACACG
frizzled3	*V. cardui*	**M13F**, forward	**TGTAAAACGACGGCCAGT**GTAAGGGAGAAGAAAGCCAC
		reverse	CAGCATTTACGGGTATCCTG
frizzled4	*V. cardui*	**M13F**, forward	**TGTAAAACGACGGCCAGT**AGCCAAAGTAATACAGCAGC
		reverse	TCAATATGGATGTTCGTCGC
frizzled2	*D. plexippus*	forward	CAGAAGTGCGTGCCAGCTTTCTTTG
		reverse	CTCCACCAGCGGCCAATATTGATGG

Table S4. PCR primer sequences for the generation of riboprobe PCR transcription templates

Antisense mRNA riboprobe	Species	Features	Oligonucleotide sequence 5' to 3'
frizzled1	*V. cardui*	forward	TTTGACTTGAAGTGGCCAAA
		**T7**, reverse	**TAATACGACTCACTATAGGG**AGCGGCCAAATGAAAATACT
frizzled2	*V. cardui*	forward	ATCCGCTCGGTCATCAAG
		**T7**, reverse	**TAATACGACTCACTATAGGG**TCACACGGCCCCCTTGAC
frizzled3	*V. cardui*	forward	GTGGCTTTCTTCTCCCTTAC
		**T7**, reverse	**TAATACGACTCACTATAGGG**TTGAGTAGGCGCGTTTAAAA
frizzled4	*V. cardui*	forward	AGAGAAAAGCAGCAGAAGTG
		**T7**, reverse	**TAATACGACTCACTATAGGG**TTGAGTAGGCGCGTTTAAAA

Development • Supplementary information

Table S5. Dicer-substrate siRNA (DsiRNA) reagents used in pupal wing electroporations for gene expression knockdowns.

siRNA target	Target Species	DsiRNA name	RNA sequence 5' to 3'<br>S : Sense (S) and Antisense (AS) strands<br>lower case: deoxyribonucleic	Injection mix
fz1	*J. coenia*	*Jc_fz1*	S: AAAAGGUAGAAGAACUGAAGCAUat	100 µM
			AS: AUAUGCUUCAGUUCUUCUACCUUUUAU	
	A. incarnata + J. coenia	*Ai+Jc_fz1*	S: AGUUGAUGGUGAUAUUUUGUCAGgt	100 µM
			AS: ACCUGACAAAAUAUCACCAUCAACUUU	
fz2	*J. coenia*	*Jc_fz2_exon1_#1*	S: ACCUCUCCGCUUGCUACUUCAUGGUC	140 µM each duplex at 70 µM
			AS: GACCAUGAAGUAGCAAGCGGAGAGGU	
		Jc_fz2_exon1_#2	S: CGGCGCUGAUGCUCAAGUACUUCAUG	
			AS: CAUGAAGUACUUGAGCAUCAGCGCCG	
Ubx	*J. coenia*	*Jc_Ubx_exon1*	S: AUUCUAUCCUUGGAUGGCCAUUGCA	140 µM each duplex at 70 µM
			AS: UGCAAUGGCCAUCCAAGGAUAGAAU	
		Jc_Ubx_exon2	S: AGACAGACCUACACUAGAUAUCAAAC	
			AS: GUUUGAUAUCUAGUGUAGGUCUGUCU	
EGFP	none	*EGFP_siRNA*	S: GGUGAUGCAACAUACGGAAAACUUAC	70 µM
			AS: GUAAGUUUUCCGUAUGUUGCAUCACC	

Table S6. Amino-acid sequences of Frizzled-family proteins in insects used in Fig. S1.

Click here to download Table S6

Development • Supplementary information
